# Sino-Australian University Partnership in Health Management Education

**DOI:** 10.3389/fpubh.2018.00251

**Published:** 2018-09-07

**Authors:** Sandra G. Leggat, Chaojie Liu, Qunhong Wu

**Affiliations:** ^1^Department of Public Health, School of Psychology and Public Health, La Trobe University, Melbourne, VIC, Australia; ^2^Department of Social Medicine, School of Public Health, Harbin Medical University, Harbin, China

**Keywords:** health management, transnational education, academic partnership, health administration, management training, global health

## Abstract

This paper outlines a successful partnership program between La Trobe University in Melbourne Australia, and Harbin Medical University in Harbin, China. These two universities have been collaborating for more than 15 years to provide a comprehensive Master of Health Administration program that adapts the Australian curriculum to meet the rapidly increasing need for qualified health services managers throughout China. This paper describes the mechanisms by which the joint programs were developed and how the two universities work together in partnership to continually improve the program components and outcomes, taking into account the significant differences in context and cultures. Since 2001, La Trobe University has enrolled about 1000 Chinese health services managers, with 721 completing a Master's degree, who are now having increasing influence on the reforms of the Chinese health care system. The partnership has enriched Australian knowledge of Chinese culture and values, as well as the Chinese health system and health policies, as evidenced by the large volume of joint publications. The profession of health management has been substantially strengthened in China, and working together, Chinese and Australian academics have had demonstrated impact on enhancing the reforms of the Chinese public health system. Further studies, with sufficient funds for data collection, are needed to evaluate the long-term impacts of transnational programs on academic and health system development in China.

## Background and rationale

The La Trobe University China Health Program (CHP) courses were developed in response to rising concerns about both the quality of care and the efficiency of service delivery in Chinese hospitals in the 1990s (1, 2). The economic and social reforms following the Third Plenum of the 11th Central Committee of the Chinese Communist Party in December 1978 aimed to transform the economic system from a socialist planned economy to a socialist market economy. In public health care, these reforms had far-reaching effects, reducing the financial contribution of the government, with the expectation that public hospitals would raise a growing portion of their operating revenue through user charges (3).

In addition, Chinese hospital managers faced many of the same pressures as hospital managers in other countries, such as ensuring access and quality for an aging population with decreasing public sector resources; improving efficiency to stretch the limited resources; and selectively implementing new, and often expensive, technologies. Despite these challenges, Chinese hospital management was seen as a part-time responsibility, not a vocation. Managers were appointed, principally from senior medical staff and generally on a part-time basis, as they retained their clinical involvement. Hospital managers were not provided with formal management training and were expected to learn on the job (4).

The lack of full-time professional hospital management was seen by many to contribute to the under-performance of Chinese hospitals (5, 6). In response, a study was conducted to identify the required competencies of Chinese hospital managers in three hospitals in a south-western province in China. Using both questionnaire survey and interviews, the researchers found that the managers felt they needed management training. The respondents specifically identified the need for skills in leadership (81%), communication (75%), financial management (62%), and the application of information technology to hospital services (34%) [(4), p. 21].

The study also confirmed that most senior public hospital managers in China saw their management involvement as part-time and short-term. The majority (69%) of the hospitals' chief executive officers (CEOs) had a medical background in the study sample, with the rest having a non-health professional background (e.g., retired military officer). They were appointed by the government for a fixed term. These medical practitioners expected to return to clinical practice after completing their period in management, suggesting that it was essential that they maintain their clinical skills through continuing clinical involvement throughout their managerial appointment (4). While this study identified shortfalls in the competence of the respondent managers, it was also concluded that this was not the sole contributor to the under-performance of the hospitals, as adverse health policy decisions were also found to have contributed to the performance of the study hospitals.

This need for formal management training provided the impetus for the development of a transnational (as defined by McBurnie and Ziguras) (7) La Trobe University China Health Program in 1996. Located in the Asia Pacific region, Australia was in a unique position to address the education needs of Chinese health managers. The University had a long running successful Master of Health Administration (MHA) program for Australian managers. The La Trobe MHA program was particularly attractive to the Chinese health managers for its practice orientated approach designed around a validated management competency framework [details about the competency framework have been published elsewhere, (8–10)]. La Trobe University (LTU) draws many Asian students. North Asia comprises mainland China, Hong Kong China, and Japan and 45% of LTU Asian students come from North Asia, with 90% of these from China.

## Development and pedagogies of the china health program

The La Trobe University CHP staff worked with a number of Chinese universities, aiming to adapt the highly successful MHA degree taught to Australian health managers to meet the needs of health managers in China. However, it was not until 2001 that the CHP program really took off, with the partnership with Harbin Medical University (HMU). Both universities enjoy a high profile in health management education and training in their respective country. La Trobe University, a comprehensive university with more than 30,000 annually enrolled students, is ranked among the top 500 universities in the world. Its MHA program is one of the biggest in Australia. In contrast, Harbin Medical University is a university specialized in medical sciences. It is relatively small with about 15,000 annually enrolled students. The Harbin training center for health services management was one of the first on-the-job training centers established by the Ministry of Health in China in the 1980s, which has now grown into a school delivering health management courses ranging from bachelor to masters and doctoral degrees.

Academic staff from both universities adapted existing MHA subjects to meet the needs of the Chinese health managers, with Faculty from both universities teaching into the program in English and Mandarin. Most subjects have flexibility about the context of written assignments, so that the academic skills each subject offers can immediately be translated to professional or personal interest areas. These subjects were designed for health managers or administrators working in hospitals, governmental bureau, and departments (health, civil affairs, social insurance, food, and drug administration, planning, financing, and other related fields), academic centers, and non-governmental organizations and consumer bodies. Students entering into the courses were required to have a tertiary undergraduate qualification equivalent to a baccalaureate and at least 3 years of working experience. There were no English language proficiency requirements because the courses were delivered in Mandarin (including interpretation from English to Chinese).

The intended learning outcomes of the MHA program are:

- to apply research principles and practices to critically appraise the health management body of knowledge and apply to managerial and leadership practice;
- to synthesize information on population needs, health care delivery systems, funding and financing arrangements, and government policy to create, revise, implement, and evaluate evidence-based strategies that enhance population health and maximize organizational potential;
- to apply project management tools and techniques underpinned by contemporary theoretical models to cultivate change;
- to evaluate and synthesize the impact of health system and health organization resource allocation decisions and apply to future resource allocation decisions;
- to identify and assemble relevant data to analyze performance to improve the operations of a health care system or organization to enhance patient care and population health outcomes;
- to develop and apply ethical evidence-based leadership skills directed to individuals and teams across diverse organizational environments;
- to effectively communicate ideas and information to different audiences and diverse settings; and
- to apply critical reflective practice that adapts responsively to the needs of different contexts and stakeholder groups.

The Chinese government, in a range of policy documents, recognized the importance of high-quality education and identified transnational partnerships as a method to rapidly boost the capabilities of Chinese universities (11). The 2003 Regulations of the People's Republic of China on Chinese-Foreign Cooperation for Running Schools outline basic requirements:

- Foreign institutions must partner with Chinese universities
- These partnerships must not seek profit as their objectives
- The basic language of instruction must be Chinese, and
- Tuition fees require government approval (12).

The CHP responded to all of these requirements, building the partnership with HMU.

The Program was designed with four educational components, comprising a Graduate Diploma, two Master degrees and health policy fora, which responded to the educational policies of the Chinese Ministry of Education. The first component (started in 2000), the Graduate Diploma in Health Services Management (GDHSM) was the first step for Chinese health managers. It was designed as an integral part of the MHA degree course. A stand-alone Graduate Diploma is not formally recognized by the Ministry of Education in China in its qualification framework. This enabled a range of students from throughout China to prove their abilities in Australian-style postgraduate study.

The next component, the Master of Health Administration (started in 2001), was approved by the Chinese Ministry of Education. The operational model of the Harbin-based MHA was adapted to meet the needs of the students. The students studied in intensive block modes (15–21 days of teaching per block), which minimized disruptions to their job functions. The block teaching was delivered face-to-face with workloads shared by academics from the two universities. All of the teaching and learning materials, including subject guidelines, lecture presentations, small exercises, recommended readings, and assessments and assignments were developed by La Trobe University with inputs from Harbin and several other Chinese universities in line with the requirements of the Australian Qualification Framework (AQF). Four La Trobe academic staff coordinated the subjects, with sessional inputs from others. They were paired with six Harbin academic staff. The Harbin teaching staff had to meet the AQF qualification requirements. They (except one) were also trained and appointed as adjunct staff of La Trobe University. The teaching and learning packages were distributed to enrolled students at the start of the courses. They were encouraged to preview the materials and do small exercises before attending face-to-face classes.

A variety of learning activities were involved in the block teaching (face-to-face), including academic and guest lectures, case studies, group discussions, policy debates, role play (for stakeholder analyses), facility (field or video) visits, and hands-on practices (e.g., Medical Director, a decision supporting software for general practice). During the teaching sessions and in assignments the students were encouraged to solve actual management problems in their workplace (for example, development of a local health network with an aim to improve vertical integration of services across several organizations or a pay for performance salary system within a hospital). The block teaching model allowed them to try new strategies and instruments in their organizations (for 3–4 months), and bring questions and feedback to the next block of training. This formed a learning-practice-reflection-change cycle (13). The Chinese health management students were keen to keep up with international trends in health system development and health services management. This course design expanded the vision of the students by exposing them to the experiences of various countries without necessarily traveling overseas.

In recognition of the demand for health management education, the initial quota of 50 MHA students was increased to accommodate 70 students. These 70 students were able to enrol at Harbin Medical University for the joint HMU/LTU MHA Program. However, more than 120 eligible applications were regularly received in Harbin every year. This limitation to 70 enrolled students meant that many applicants would not be offered a place to study the MHA in China.

**Figure 1:**
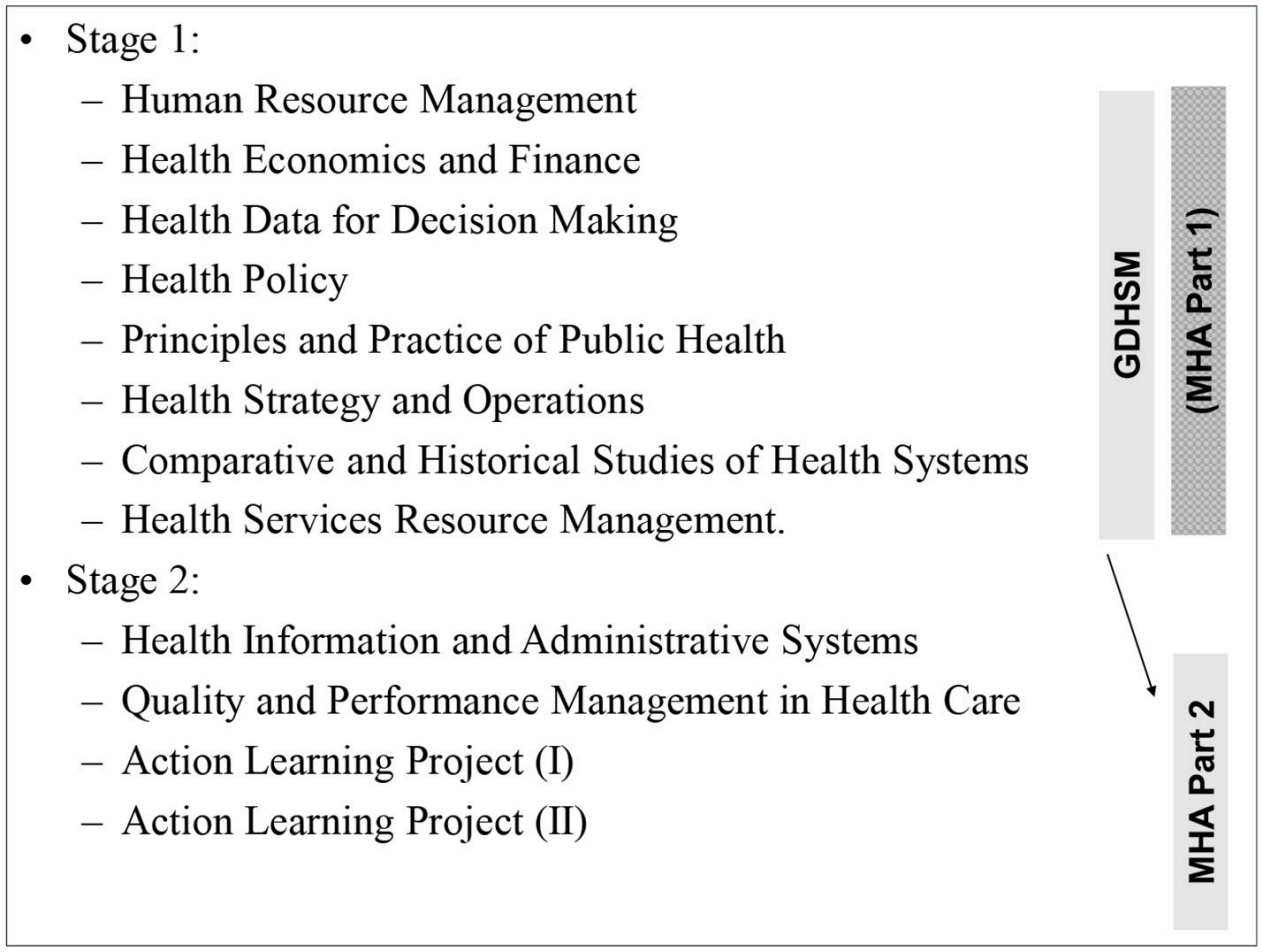
Standard sequence of study and course enrolments offered as part of the CHP.

The Graduate Diploma in Health Services Management (GDHSM) and the MHA form an integrated program, outlined in Figure 1. A full MHA includes 11 subjects (Figure 1), with a total of 180 credit points (10 subjects accrue 15 credit points, one subject accrues 30 points).

This led to the development of the third component, a 1-year full-time MHA (120 credit points) offered onshore (started in 2001) at La Trobe University in Melbourne, Australia. It was expected that this onshore MHA would accommodate students who had completed training in China equivalent to the Graduate Certificate (60 credit points equivalence) or the GDHSM, but who were not accepted to the highly competitive partnership MHA in Harbin. The onshore Australian operations also enabled an expansion of La Trobe MHA partnerships in China beyond Harbin Medical University.

In theory, this worked for a number of years, but La Trobe University eventually closed this onshore Program, as it was difficult to obtain sufficient numbers of Chinese students to study in Australia to be viable. There were a number of reasons for the difficulties in building the capacity of the onshore Program. The first was that most of the applicants were in health care management positions and it was difficult for them to leave their jobs, and the country for an extended period of time to study. Getting permission to leave China and obtaining an Australian visa was often difficult, as these applicants were not the typical undergraduate students looking for a study visa for Australia. Finally, various public health events, such as severe acute respiratory syndrome (SARS) and climate events, such as the earthquakes, resulted in even those students with approved study visas being recalled to their various roles in the public health system in China.

The Chinese governmental approved fee levels for the HMU/LTU MHA Program (increasing over the years), which enabled a financial balance through sharing costs (including both academic and administrative costs) and revenues between the two universities, but with limited profits. This lack of profit incentive was consistent with the 2003 Regulations of the People's Republic of China on Chinese-Foreign Cooperation for Running Schools. As such many of the impacts of the Program were in areas other than financial, for example, the fourth educational component of the CHP that aimed to influence health policy and health system development in China. The La Trobe CHP successfully organized several Sino-Australia Fora on Health System Reform in Beijing and Harbin, with funding support from the Australian China Council (ACC) and Chinese funding bodies. These fora attracted tremendous interest from both China and Australia (e.g., more than 500 participants in the 2010 forum in Beijing) (14). Participants included the La Trobe CHP alumni, hospital managers, community health managers, health officials, and health research academics from both countries, as well as representatives from international agencies connected with China, such as the World Health Organization, the Australian Embassy, AusAID, and its HIV/AIDS Facility (CHHAF), the World Bank, and the UK Department for International Development.

Through these fora, the La Trobe CHP also coordinated study tours for Australian health managers to China and for Chinese health managers to Australia. In a broad sense, Australia and China face similar health policy challenges despite the differences between the two countries. Both countries are working to reduce gaps in health services accessibility and in health outcomes between rich and poor, urban and rural, and indigenous and non-indigenous people. China can learn from Australia's experiences in the past decades, in particular, the development of a universally accessible Medicare system. Meanwhile, Australians can also benefit from the reframing which happens when they seek to make sense of a familiar policy questions in very different settings. These fora attracted interest from a wide range of public media, including Chinese Central Television, the New Peking Newspaper, the Morning Newspaper, the Health Newspaper, the Beijing Youth Newspaper, Xinhua Times, and Xinhua News Agency.

The CHP MHA is derived from and is consistent with the La Trobe University MHA degree. The MHA meets both the Australian Tertiary Education Quality and Standards Agency (TEQSA) requirements for a Master-level qualification (15) and the competency requirements for accreditation by the Australasian College of Health Service Management (16). The teaching program was both strengthened and supported by a comprehensive collaborative research program as well.

## The china health program outcomes

**Figure 2:**
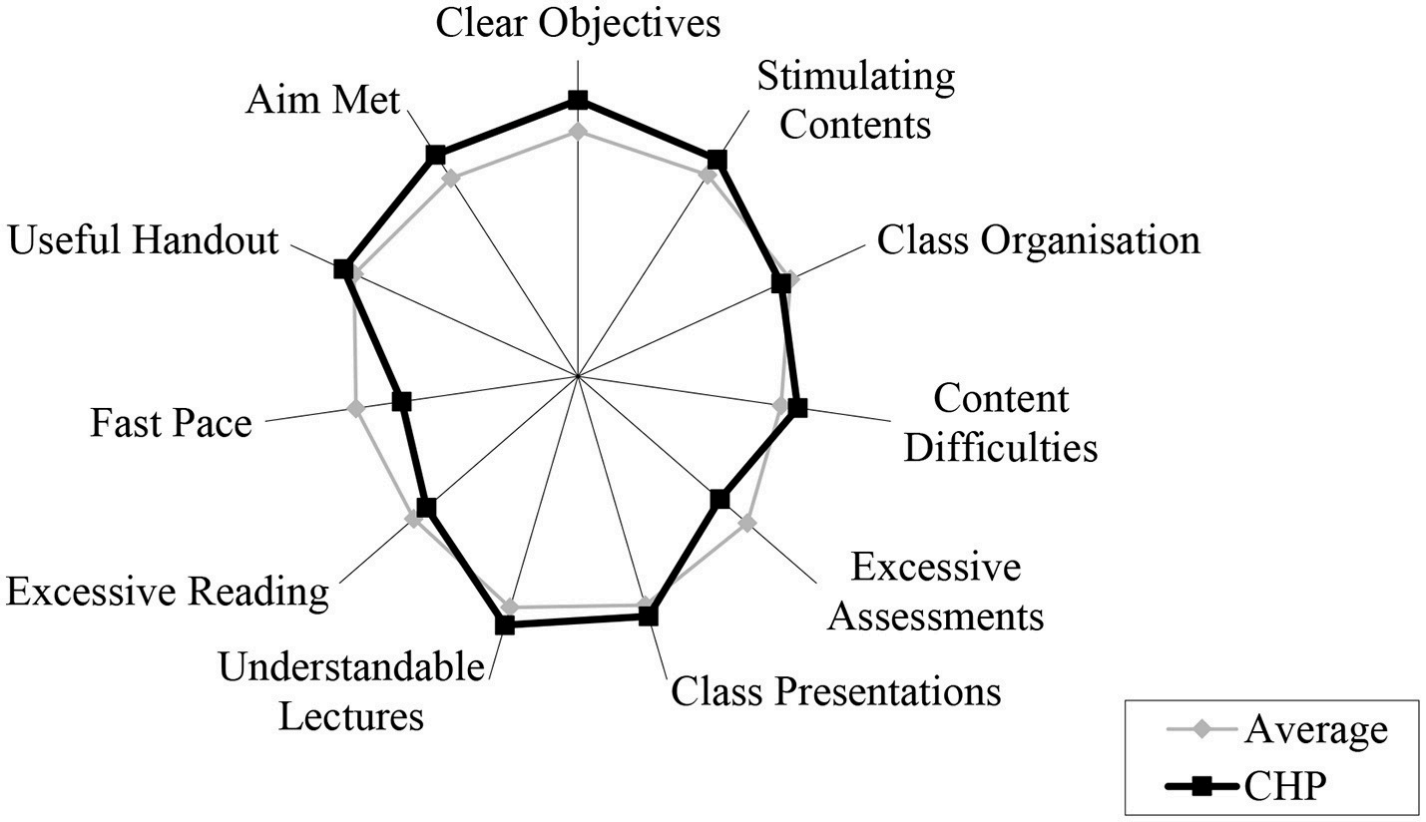
Student feedback on health courses at La Trobe University (2002). Note: a higher score (longer distance from the center) indicates more positive feedback.

Given the tenure of the CHP, there has been substantial internal evaluation, as well as external recognition of the program. The CHP aims to support its students, graduates, and alumni to make a real difference to their management practice and the performance of their organizations and the Chinese health system. Overall, students expressed a higher level of satisfaction for the CHP courses compared to the average of health courses at La Trobe University, according to a sampling survey in 2002 using a validated instrument developed by the University. The CHP students were more likely to believe that the courses were useful to help them achieve the relevant aim and objectives despite some concerns about the high study loads and fast pace of learning (Figure 2). The annual student feedback survey showed that CHP students consistently rated the MHA subjects high over the years, with an average score of over 4.5 out of a possible 5.

A questionnaire survey in 2005 collected qualitative data in relation to the achievements of 63 students who had completed the CHP MHA. The results indicated that the students were able to apply what they learned in the MHA to their workplaces (17). The respondents specifically identified that the MHA helped them develop the knowledge and skills to:

- improve the safety and quality of patient care,
- build a learning organization by taking advantage of modern information technology,
- balance the needs of various stakeholders for the sustainable development of their organizations,
- nurture a high-performance work system,
- improve operations management by learning from other industries (such as lean thinking and six sigma), and
- participate in policy dialogues and health system reform.

Adding even more value, about half of the respondents published peer-reviewed articles in policy or management journals, and 23% acknowledged the role of their learning achievements on career promotions and cited examples of grants, projects, awards, and publications as a result of the study (17).

There is evidence that the La Trobe China Health Program has started showing long-term effects on students' participation in the health system reform in China. For example, case mix or activity-based funding has been one of the areas with relevant application. The theory, concept and applications of Diagnostic Related Groups (DRGs) were introduced at the start of the MHA course in 2000/2001, which attracted immediate interest from the students (18). Later on, a group of MHA students established a Chinese version of DRGs (19) with technical input from La Trobe University academics based on a doctoral research project on casemix classifications in acute hospital settings in China (20). More recently, La Trobe CHP has been invited to participate in a national pilot project on casemix funding for hospitals (21). This project is led by a student who graduated from the first cohort of the Harbin joint MHA program. The CHP role includes a capacity assessment of the pilot cities, training of policy makers, and health services managers from the central government and the pilot cities, and consultation services to the project team. Broader evaluation of the health system impact of this transnational initiative would require a comprehensive assessment that was not possible within the budget of the CHP. Further research and evaluation is an essential next step.

The La Trobe CHP is able to make these documented achievements for several reasons. The first is that the students value the complementary nature of teaching delivered by the Chinese and Australian teachers. According to the cultural tradition, Chinese students respect and favor a teacher-led systematic approach in learning (22). This approach usually starts with an overarching theoretical logic explanation before details of various management strategies are discussed. While the presentations made by the Australian lecturers were often considered novel and innovative, the students found that compared to Chinese pedagogy, the Australian content was fragmented. The Chinese partner lecturers were well positioned to help the students comprehend what they were taught in a more culturally appropriate and less cognitively challenging way. A close working relationship was built between Australian and Chinese academics using a capacity building model to enhance teaching and learning skills.

Second, the bilingual teaching capacity played a critical role in bridging the gaps and potential mismatches between the western management theories and the Chinese context. The course adopted a co-teaching model involving monolingual teachers (English only or Chinese only) working closely with bilingual teachers (with Chinese as the native language). The bilingual teachers understood western management theory and practice, as well as the Chinese context. This enabled the bilingual interpreters to move well beyond translation, facilitating student learning of the content within the Chinese context, and providing real world examples. Over time the bilingual lecturers also developed an excellent understanding of the La Trobe University teaching pedagogy. Regular teaching and planning workshops enabled Australian and Chinese teachers to share perspectives on curriculum and pedagogy.

Third, the students requested a set of unified textbooks, which were deemed critical for a systematic and logic approach to learning. Although it is difficult to keep textbooks updated in a timely manner, the La Trobe CHP compiled several bilingual [e.g., Health Policy in and for China (23); Working with Information (24); Project Management in Health, and Community Services (25)] and monolingual textbooks [e.g., Health Care in Australia (Chinese) (26); Leading and Managing Health Services (in English) (27); Health Human Resource Management (in Chinese) (28)] while still maintaining an updated package of reading materials. The teaching and learning materials were packaged in electronic format and enrolled students could get access to these materials both online and offline (through CDs/USB sticks). The completion of the textbooks provided additional opportunities for collaboration and cooperation between the HMU and LTU academic staff. Both universities took great pride in these accomplishments, with a large scale celebratory book launch involving senior government officials from both Australia and China.

Fourth, similar to the domestic Australian version, the course recognized that the senior managers enrolled in the courses had a wealth of experience, judgement and knowledge that comprised a key resource in the courses. The mode of delivery ensured that students reflected on and learned from their own experience and from each other, through the sharing of experience, strategies, and collective reflection on practice.

Finally, the teaching was supported by relevant research. The LTU CHP staff engaged in research into the health care system in China in collaboration with academics from the Chinese universities and the CHP alumni. The research activities enabled Australian staff to develop a better understanding of the Chinese health system and the challenges the students were confronting. This is evidenced by 11 monographs/textbooks and an impressive 173 peer-reviewed publications produced by the CHP staff about the Chinese health system and health reforms published in both English and the Chinese language. Topics have included patient satisfaction (29–31), medications policy and practice (32–40), patient safety and organizational safety culture (41, 42), health reform (35, 43–50), health insurance (50–52), emergency response (53–56), and human resource management (3).

The achievements of the CHP have been widely recognized in both China and Australia. For example, the CHP Master of Health Administration program (offshore in Harbin) was commended by the Australian University Quality Agency (AUQA) as a potential “Jewel in the Crown” of La Trobe's international activities and was chosen for inclusion in the AUQA Good Practice Database. Further, the CHP won an award in 2006 from the Ministry of Education of China for educational innovation and the inaugural Victorian International Education Award for *Excellence in Innovation in International Education* 2013 (https://www.latrobe.edu.au/news/articles/2013/release/recognition-for-international-education). This was a government award provided by the State of Victoria in Australia as part of its international strategy (http://www.invest.vic.gov.au/news-and-events/2013/oct/2013-10-28-victorias-new-international-education-strategy-for-the-asia-pacific-region).

## Lessons learned along the way

As a host University there is a temptation to assume the role of the “expert.” However we learned early on that being the expert was not compatible with the public health approach to capacity building that recognizes the expertise, values and cultures of our partners. Australian and Chinese national cultures would fit at opposite ends of many cultural dimensions, such as individualism and collectivism (57), and specific vs. diffuse (58). The recognition of these differences with incorporation into the curriculum was even more important than addressing different characteristics of the health care systems. “A method born of one culture may be adapted to another only when relevant cultural differences are rigorously considered” [(59), p. 417].

For example, while many Australian universities, particularly in health education, have implemented problem-based or enquiry-based learning (60). Kee and Wong (22) found that Chinese students learned better when the information was given by the teacher and did less well when forced to discover the answers for themselves. The program was structured to ensure students were provided with the foundational information, and the application of this information in the development of management competencies were facilitated through a reflective learning approach (13).

With support from teachers, students were encouraged to enter into “deep learning.” This usually started with an introduction of facts, practices, and their underlining principles by teachers, followed by student reflections on examples (either successful or failed examples) in China through group discussions. The students were then challenged to discuss why a particular theory or principle may or may not work within the Chinese context. A clear message was sent to students in many assignments: they were welcomed to challenge teachers, and teachers were more than happy to see arguments and thoughts that deviated from classroom teaching and textbooks. In the capstone action learning project, students had to discuss why their project activities achieved or failed to achieve intended goals. Recognizing and learning from failure comprised an important part of the reflective learning process.

As outlined by Phuong-Mai and colleagues (59), a joint curriculum spanning cultures should not take “an either-or” approach, but should be a compromise that is superior to the initial product. Asian students want to be sure that what they are learning in a transnational education program is easily adapted to their local context and not generic education that is difficult to apply (61). As a result, the Australian academic staff learned as much about the Chinese health system, Chinese cultures and values as the Chinese students and staff learned about Australia and western management practices.

Developing a targeted culturally sensitive curriculum is made more difficult by the increasing economic pressures for the internationalization of Australian university programs. Schapper and Mayson (62) have commented on the very real tensions between the need for a tertiary education product that is acceptable to students around the world, while at the same time ensuring these programs can meet the diverse localized needs of specific student groups. They stress the need to recognize and support the “important link between academics' research activities and the contribution these activities make to the pedagogical soundness of the courses we offer students in an internationalized context” [(62), p. 202]. These authors suggest that there is an economic temptation to “water down” the curriculum to make it acceptable to a range of different contexts. Our experience suggests that the CHP would not have been as successful in equipping the graduates to make changes in their own health system without the partnership approach that was used.

Similarly, many of the teaching tools used in the Australian context were not able to be easily adapted to meet the needs of the Chinese students. Some of the Chinese restrictions on social media and the difficulties in scaling organizational intranet firewalls provided some limitations, which were overcome by working in partnership to ensure the final goals were achieved.

Our final learning was the need to plan for broad longitudinal evaluation when planning the programs. Rarely do teaching programs contain sufficient budget for the necessary comprehensive evaluation and this needs to be a focus of future transnational health management education programs.

## Conclusions

The La Trobe University and Harbin Medical University health administration teaching and research partnership has developed over decades for the benefit of both universities and faculty members in Australia and China. The partnership has enriched Australian knowledge of Chinese culture and values, as well as the Chinese health system and health policies. The profession of health management has been substantially strengthened in China, and working together, Chinese and Australian academics have had demonstrated impact on enhancing the reforms of the Chinese public health system.

Some key themes can be extracted from the successful operations of the Program: (1) mutual understanding: the Australian participants were as keen as the Chinese participants to learn from their counterparts; (2) evidence-based learning: research was essential in the curriculum development in order to meet the practical needs of students; (3) complementary skills and responsibilities: the two universities shared academic and administrative resources in a complementary way; (4) faculty development: the two universities shared a common goal in internationalization; (5) engagement and participation: academic staff supported students to participate in health reforms; and (6) good communication and coordination: bilingual academics played a critical role in the governance and operations management of the Program. While these are essential factors for future program development, the important message is that partnerships may evolve in different ways depending on the culture and values and needs of both partners. It is an evolving, learning experience for both partners.

## Author contributions

All authors listed have made a substantial, direct and intellectual contribution to the work, and approved it for publication.

### Conflict of interest statement

The authors declare that the research was conducted in the absence of any commercial or financial relationships that could be construed as a potential conflict of interest.
